# Medical help-seeking intentions among patients with early Alzheimer’s disease

**DOI:** 10.3389/fpsyt.2023.1290002

**Published:** 2023-12-19

**Authors:** Alberto Villarejo-Galende, Elena García-Arcelay, Gerard Piñol-Ripoll, Antonio del Olmo-Rodríguez, Félix Viñuela, Mercè Boada, Emilio Franco-Macías, Almudena Ibañez de la Peña, Mario Riverol, Albert Puig-Pijoan, Pedro Abizanda-Soler, Rafael Arroyo, Miquel Baquero-Toledo, Inmaculada Feria-Vilar, Mircea Balasa, Ángel Berbel, Eloy Rodríguez-Rodríguez, Alba Vieira-Campos, Guillermo Garcia-Ribas, Silvia Rodrigo-Herrero, Alberto Lleó, Jorge Maurino

**Affiliations:** ^1^Department of Neurology, Hospital Universitario 12 de Octubre, Instituto de Investigación Imas12, Universidad Complutense de Madrid, Madrid, Spain; ^2^Medical Department, Roche Farma, Madrid, Spain; ^3^Cognitive Disorders Unit, Hospital Universitari Santa Maria de Lleida, Lleida, Spain; ^4^Department of Neurology, Hospital Universitario Dr. Peset, Valencia, Spain; ^5^Cognitive Impairment Unit, Hospital Universitario Virgen Macarena, Seville, Spain; ^6^Ace Alzheimer Center Barcelona, Barcelona, Spain; ^7^Dementia Unit, Department of Neurology, Hospital Universitario Virgen del Rocío, Seville, Spain; ^8^Policlínica Guipúzcoa, San Sebastián, Spain; ^9^Department of Neurology, Clínica Universidad de Navarra, University of Navarre, Pamplona, Spain; ^10^Cognitive Impairment and Movement Disorders Unit, Department of Neurology, Hospital del Mar, Barcelona, Spain; ^11^Department of Geriatrics, Complejo Hospitalario Universitario de Albacete, Albacete, Spain; ^12^Department of Neurology, Hospital Universitario Quirónsalud, Madrid, Spain; ^13^Grup d'Investigació en Malaltia d'Alzheimer, Department of Neurology, Hospital Universitari i Politècnic La Fe, Valencia, Spain; ^14^Department of Neurology, Complejo Hospitalario Universitario de Albacete, Albacete, Spain; ^15^Unit of Alzheimer's Disease and Other Cognitive Disorders, Hospital Clínic, Barcelona, Spain; ^16^Department of Neurology, Hospital Central de la Cruz Roja, Madrid, Spain; ^17^Department of Neurology, Hospital Universitario de Marqués de Valdecilla, IDIVAL, University of Cantabria, Santander, Spain; ^18^Department of Neurology, Hospital Universitario de La Princesa, Madrid, Spain; ^19^Department of Neurology, Hospital Universitario Ramón y Cajal, Madrid, Spain; ^20^Memory Unit, Department of Neurology, Hospital Universitario Juan Ramón Jiménez, Huelva, Spain; ^21^Department of Neurology, Hospital de la Santa Creu i Sant Pau, Barcelona, Spain

**Keywords:** Alzheimer’s disease, help-seeking, early diagnosis, illness representation, awareness

## Abstract

**Background:**

Limited information is available on the active process of seeking medical help in patients with Alzheimer’s disease (AD) at early stages. The aim of this study was to assess the phenomenon of medical help-seeking in early AD and to identify associated factors.

**Methods:**

A multicenter, non-interventional study was conducted including patients of 50–90 years of age with prodromal or mild AD (National Institute on Aging/Alzheimer’s Association criteria), a Mini-Mental State Examination (MMSE) score ≥ 22, and a Clinical Dementia Rating-Global score (CDR-GS) of 0.5–1.0. A multivariate logistic regression analysis was conducted.

**Results:**

A total of 149 patients were included. Mean age (SD) was 72.3 (7.0) years, 50.3% were female, and 87.2% had a CDR-GS score of 0.5. Mean disease duration was 1.4 (1.8) years. Ninety-four (63.1%) patients sought medical help, mostly from neurologists. Patients with help-seeking intentions were mostly female (60.6%) with a CDR-GS score of 0.5 (91.5%) and had a greater awareness of diagnosis, poorer quality of life, more depressive symptoms, and a more severe perception of their condition than their counterparts. Lack of help-seeking intentions was associated with male sex (*p* = 0.003), fewer years of education (*p* = 0.005), a low awareness of diagnosis (*p* = 0.005), and a low emotional consequence of the condition (*p* = 0.016).

**Conclusion:**

Understanding the phenomenon of active medical help-seeking may facilitate the design of specific strategies to improve the detection of cognitive impairment, especially in patients with a lower level of educational attainment and poor awareness of their condition.

## 1 Introduction

Alzheimer’s disease (AD) is a prevalent chronic neurodegenerative disorder with a devastating impact on the quality of life of patients and their families (1). The current management landscape is rapidly evolving with the emergence of specific diagnostic biomarkers and the recent approval of therapies targeting the beta-amyloid protein in patients with prodromal or mild AD (2–4).

A specific AD diagnosis at early stages enables patients and their families to be able to make decisions and plan their future and also starting pharmacological and non-pharmacological therapies to delay cognitive decline (5). However, a timely diagnosis is still uncommon due to several factors (6). Knowledge of the disease in society is still limited, particularly in terms of prodromal symptoms despite different public awareness campaigns (7, 8). Misconceptions, public stigma, and lack of effective treatments can also act as barriers to diagnosis as symptoms appear (9, 10).

Timely diagnosis of AD can be improved by encouraging patients to seek medical help early (11). AD patients are able to describe their problems, experiences and preferences at different stages of the disease, although anosognosia is a very prevalent feature (11–13). The educational background, family history of cognitive impairment, and the identification of symptoms and their affective impact were associated with help-seeking intentions among patients facing a diagnosis of AD (14–16). However, the phenomenon of active medical help-seeking in the early stages of AD and its associated factors remains underexplored with a number of conceptual and methodological limitations, including the lack of standardized instruments for its assessment (11). In addition, most previous research has focused on the role of patients’ family and friends in identifying symptoms and seeking help from the healthcare system (11, 17, 18). The aim of this study was to assess help-seeking intentions in patients with prodromal or mild AD using a battery of patient-reported and physician-rated measures.

## 2 Methods

A non-interventional, cross-sectional study was conducted at 21 hospital-based memory clinics in Spain. Patients between 50–90 years old with a diagnosis of prodromal or mild AD according to the National Institute on Aging/Alzheimer’s Association criteria, a Mini-Mental State Examination (MMSE) score ≥ 22, and a Clinical Dementia Rating-Global score (CDR-GS) of 0.5–1.0 were invited to participate in the study and evaluated in a single session in the context of their regular follow-up visits in their memory units (19–21). The study was approved by the research ethics board of Hospital de la Santa Creu i Sant Pau (Barcelona, Spain). Written informed consent was obtained from all participants. Patients were recruited between February and June 2021.

Two self-report questions were used for help-seeking assessment based on previous studies: “Did you seek medical help when you noticed cognitive problems (memory loss, disorientation or other symptoms)?” and “From what sources? (11). The Quality of Life in Alzheimer Disease Scale (QoL-AD), AD Assessment Scale-Cognition-Subscale 13 (ADAS-Cog13), Brief Illness Perception Questionnaire (B-IPQ), Representations and Adjustment to Dementia Index (RADIX), Beck Depression Inventory – Fast Screen (BDI-FS), Stigma Scale for Chronic Illness (SSCI-8), and General Self-Efficacy Scale (GSES) were administered to gather information on quality of life, cognition, illness representation, mood, stigmatization, and self-efficacy, respectively (22–28). Table 1 shows details of patient-reported and clinician-rated outcome measures administered.

**Table 1:** Outcome measures.

Outcome	Measure	Scoring and interpretation	Range
Cognition	MMSE	It is an 11-question measurement to assess cognitive performance. A cut-off of ≤23 is used to identify patients with cognitive problems.	0–30
	ADAS-Cog13	It is a 13-task instrument to assess the level of cognitive dysfunction in patients with predementia and Alzheimer’s disease. It includes both subject-completed tests and observer-based assessments. Higher scores indicate greater dysfunction.	0–85
Self-efficacy	GSES (self-rated)	The GSES is a 10-item instrument to assess whether a person can face adversity in different domains of functioning. Each item is rated using a four-point scale ranging from 1 (not at all true) to 4 (exactly true). Higher scores indicate higher levels of an optimistic self-belief.	10–40
Mood	BDI-FS (self-rated)	The BDI-FS is a seven-item questionnaire to assess depressive mood. Each item is rated on a four-point scale (no symptoms to severe symptoms). A cutoff ≥4 indicates the presence of depressive symptoms.	0–21
Quality of life	Qol-AD (self-rated)	The QoL-AD assesses quality of life of patients with a diagnosis of AD. It consists of 13 items rated on a four-point scale (from poor to excellent). Higher scores indicate better quality of life.	13–52
Disease awareness	RADIX (self-rated)	The RADIX assesses patients′ understanding of their condition and its consequences. Identity and cause are open questions. Awareness of AD diagnosis was defined when participants specifically used the word Alzheimer to refer to the diagnosis of their condition.	Yes-No
Illness representation	B-IPQ (self-rated)	The B-IPQ assesses cognitive and emotional illness representations. It consists of eight items rated on a scale from 0 (minimum) to 10 (maximum). Higher scores indicate a threatening illness perception.	0–80
	RADIX (self-rated)	The RADIX assesses patients′ understanding of their condition and its consequences. For practical and emotional consequences, responses to the questions are rated on a four-point scale (from strongly disagree to strongly agree). Higher scores indicate greater negative consequences.	0–4
Stigma	SSCI-8 (self-rated)	The SSCI-8 assesses internalized and experienced stigma across neurological conditions. Each item is rated on a 5-point Likert scale from 1 (never) to 5 (always). Higher scores indicate the presence of stigmatization.	8–40

AD, Alzheimer’s disease; ADAS-Cog, Alzheimer’s Disease Assessment Scale-Cognitive; BDI-FS, Beck Depression-Fast Screen; B-IPQ, Brief Illness Perception Questionnaire; CDR-GS, Clinical Dementia Rating-Global Score; GSES, Global Self-Efficacy Scale; MMSE, Mini-Mental State Examination; QoL-AD, Quality of Life in Alzheimer’s Disease; RADIX, Representations and Adjustment to Dementia Index; SD, Standard deviation; SSCI-8, Stigma Scale for Chronic Illness (SSCI-8).

Demographic and clinical characteristics were summarized using frequencies (percentages) and mean (standard deviation) or median (interquartile range) as appropriate. Value of *p*s <0.05 were considered statistically significant. A multivariate logistic regression analysis was conducted using a stepwise selection method to assess the association between lack of help-seeking intentions and demographic and clinical characteristics as well as patients’ perspectives. Variables with a value of *p*s <0.2 in the preliminary bivariate analysis were included as candidate variables in the model.

## 3 Results

A total of 149 patients were included. Mean age (SD) was 72.3 (7.0) years, 50.3% were female, and 87.2% had a CDR-GS score of 0.5. Mean disease duration was 1.4 (1.8) years. Main socio-demographic and clinical characteristics are shown in Table 2.

**Table 2:** Description of participants according to medical help-seeking intentions.

	Help-seekers *N* = 94	Non help-seekers *N* = 55	Total *N* = 149	Value of *p*
Age, years, mean (SD)	72.0 (7.5)	72.7 (6.1)	72.3 (7.0)	0.556^*^
Sex, female, *n* (%)	57 (60.6)	17 (31.5)	75 (50.3)	0.001^**^
Education, years, mean (SD)	14.4 (11.8)	10.6 (4.6)	13.1 (9.9)	0.034*
Disease duration, years, mean (SD)	0.9 (1.4)	0.8 (1.16)	1.4 (1.8)	0.592^*^
CDR-GS score of 0.5, *n* (%)	86 (91.5)	43 (79.6)	130 (87.2)	0.044^**^
MMSE score, mean (SD)	24.5 (2.1)	24.7 (2.2)	24.6 (2.1)	0.685^*^
ADAS-Cog13 score, mean (SD)	23.9 (5.3)	25.4 (5.1)	24.4 (5.2)	0.109^*^
QoL-AD score, mean (SD)	37.1 (4.6)	39.3 (4.1)	37.9 (4.5)	0.004^*^
GSES score, mean (SD)	29.6 (6.2)	30.6 (6.6)	30.0 (6.3)	0.357^*^
BDI-FS score, mean (SD)	2.4 (2.2)	1.6 (2.2)	2.1 (2.2)	0.037^*^
BDI-FS score ≥ 4, *n* (%)	25 (26.6)	7 (12.9)	33 (22.1)	0.062^**^
SSCI-8 score, mean (SD)	9.4 (2.3)	8.7 (1.4)	2.1 (2.2)	0.050^*^
B-IPQ score, mean (SD)	39.5 (11.3)	32.9 (9.8)	37.2 (11.2)	0.0004^*^
Awareness of AD diagnosis (RADIX^t^), n (%)	52 (55.3)	14 (25.4)	66 (45.2)	0.001^**^
Emotional consequences RADIX score, mean (SD)	2.3 (0.8)	1.9 (0.7)	2.2 (0.8)	0.005^*^
Practical consequences RADIX score, mean (SD)	1.8 (0.6)	1.7 (0.6)	1.8 (0.6)	0.064^*^

AD, Alzheimer’s disease; ADAS-Cog, Alzheimer’s Disease Assessment Scale-Cognitive; BDI-FS, Beck Depression-Fast Screen; B-IPQ, Brief Illness Perception Questionnaire; CDR-GS, Clinical Dementia Rating-Global Score; GSES, Global Self-Efficacy Scale; MMSE, Mini-Mental State Examination; QoL-AD, Quality of Life in Alzheimer’s Disease; RADIX, Representations and Adjustment to Dementia Index; SD, Standard deviation; SSCI-8, Stigma Scale for Chronic Illness (SSCI-8). ^t^N = 146. ^*^T-test/ANOVA; **Fisher’s test. Bold values mean that the *ps* are statistically significant.

Ninety-four (63.1%) reported that they sought medical attention when they realized their cognitive symptoms, mostly from neurologists (54.9%) and general practitioners (28.7%). Patients with help-seeking intentions were mostly female with a CDR-GS score of 0.5 and had a higher number of years of education, a greater awareness of AD diagnosis, more depressive symptoms, poorer quality of life, a more severe perception of their disease, and higher levels of emotional consequences than their counterparts (Table 2). No differences in cognitive assessments were found between both groups.

Lack of help-seeking intentions was associated with male sex (*p* = 0.003), fewer years of education (*p* = 0.005), a low awareness of diagnosis (*p* = 0.005), and a higher degree of emotional consequences (*p* = 0.016) in the multivariate analysis after adjustment for confounders (Table 3).

**Table 3:** Lack of medical help-seeking intentions: bivariate and multivariate logistic regression analysis.

	Bivariate		Multivariate*	
	Value of *p*	OR	95% CI	Value of *p*
Age, years	0.556			
Sex, female	0.001	0.27	0.12–0.64	0.003
Education	0.034	0.88	0.80–0.96	0.005
Disease duration	0.592			
MMSE score	0.685			
ADAS-Cog13 score	0.109			
CDR-GS score	0.044			
QoL-AD score	0.004			
B-IPQ score	0.0004			
GSES score	0.357			
BDI-FS score	0.037			
Awareness of AD diagnosis (RADIX)	0.001	0.29	0.12–0.70	0.005
SSCI-8 score	0.050			
Emotional consequences RADIX score	0.005	0.48	0.27–0.90	0.016
Practical consequences RADIX score	0.064			

AD, Alzheimer’s disease; ADAS-Cog, Alzheimer’s Disease Assessment Scale-Cognitive; BDI-FS, Beck Depression-Fast Screen; B-IPQ, Brief Illness Perception Questionnaire; CDR-GS, Clinical Dementia Rating-Global Score; CI, Confidence interval; GSES, Global Self-Efficacy Scale; MMSE, Mini-Mental State Examination; OR, Odds ratio; QoL-AD, Quality of Life in Alzheimer’s Disease; RADIX, Representations and Adjustment to Dementia Index; SD, Standard deviation; SSCI-8, Stigma Scale for Chronic Illness (SSCI-8). ^*^Variables with a *p*-values < 0.2 in the bivariate analysis were included as candidate variables in the model.

## 4 Discussion

Seeking medical help is an active process that includes identifying and becoming aware of the health problem, identifying the available resources needed to deal with it, and the willingness to disclose the problem with others (10, 11). Intentions to seek medical help have been studied in different diseases including cognitive disorders, where a prevalence of 2.6–18.6% has been found in adults over 60 years with memory problems (10).

Timely medical help-seeking is crucial in patients with AD, especially after the approval of new disease-modifying treatments that can delay cognitive decline at earlier stages of this condition (5, 29). However, no previous studies have examined this aspect in patients with prodromal or mild AD diagnosed with CSF biomarkers or amyloid PET. In our study, medical help-seeking was a frequent phenomenon in a sample of patients with early AD. This behavior was found more commonly among participants with disease awareness, poor quality of life, depressive symptoms, and a perception of threatening illness.

Patients’ beliefs and expectations about a disease influence their emotional reactions and coping resources (30). Awareness of cognitive and functional deficits and subjective perception of the disease may play a crucial role in the intention to seek medical help (31, 32). Werner et al. found that poor dementia-related knowledge and stigmatic beliefs were the main barriers in a systematic review of 48 studies addressing help-seeking for cognitive impairment (11). A perception of threatening illness positively predicted help-seeking intentions for cognitive impairment in a sample of 250 people older than 50 years participating in an online survey in the US (14). The main predictors of help-seeking intentions for an early dementia diagnosis were disease-related knowledge and belief about whether the majority of people approve or disapprove of this behavior in a sample of adults aged between 50 and 69 years in Ireland (17). People were more likely to seek help if they felt supported by family, friends and healthcare professionals. Gigi and Papirowitz stated that patients with mild cognitive impairment who seek professional help were characterized with intact awareness of their cognitive and emotional state (31). They had a history of subjective memory complaints, attributed their cognitive deficits to a biomedical cause, and reported higher levels of anxiety, depression and concern about their perceived cognitive deficits compared to those who did not seek help. Interestingly, no differences were found in objective memory performance between help-seekers and non-help-seekers (31). The findings of our study support the same conclusion. It is possible that awareness of cognitive deficits plays a more important role in determining which individuals seek medical help.

In addition, we found that predictors of lack of intention to seek help in our study were male sex, fewer years of education, lack of awareness of cognitive problems and their low emotional impact. These findings underline the need to continue to promote AD awareness campaigns in the general population so that people with cognitive impairment who have a low educational level and poor disease awareness can be recognized early by their family and friends and seek prompt medical help.

Our study has several limitations. First, we did not collect information on how many patients were excluded and for what reasons. A selection bias may have influenced the prevalence of help-seeking intentions as more motivated or cooperative patients may have been more likely to choose to participate in the study. Second, the cross-sectional study design limits the ability to establish causal relationships between the factors assessed and help-seeking. Third, MMSE scores were not adjusted for educational level. Finally, there is a lack of information collected on different factors known to be related to help-seeking intentions, such as personal exposure to AD, the perception of social support, disease knowledge, motivational aspects, and cultural and race factors (13, 33–35).

## 5 Conclusion

Medical help-seeking was a frequent phenomenon in a sample of patients with early AD. Awareness of symptoms rather than memory performance, seem to play a crucial role in this phenomenon.

Understanding these associated factors may facilitate the design of specific strategies to avoid delay in help-seeking intentions by patients with cognitive impairment and limited awareness of their condition. Further studies with a longitudinal design and in other countries are needed to understand the full spectrum of mechanisms involved in help-seeking among patients with early AD.

## Data availability statement

The raw data supporting the conclusions of this article will be made available by the authors, without undue reservation.

## Ethics statement

The studies involving humans were approved by Hospital de la Santa Creu i Sant Pau (Barcelona, Spain). The studies were conducted in accordance with the local legislation and institutional requirements. The participants provided their written informed consent to participate in this study.

## Author contributions

AV-G: Conceptualization, Investigation, Methodology, Supervision, Writing – original draft. EG-A: Conceptualization, Formal analysis, Methodology, Writing – review & editing. GP-R: Data curation, Investigation, Validation, Writing – review & editing. AO-R: Data curation, Investigation, Validation, Writing – review & editing. FV: Data curation, Investigation, Validation, Writing – review & editing. MeB: Data curation, Investigation, Validation, Writing – review & editing. EF-M: Data curation, Investigation, Validation, Writing – review & editing. AI: Data curation, Investigation, Validation, Writing – review & editing. MR: Data curation, Investigation, Validation, Writing – review & editing. AP-P: Data curation, Investigation, Validation, Writing – review & editing. PA-S: Data curation, Investigation, Validation, Writing – review & editing. RA: Data curation, Investigation, Validation, Writing – review & editing. MB-T: Data curation, Investigation, Validation, Writing – review & editing. IF-V: Data curation, Investigation, Validation, Writing – review & editing. MiB: Data curation, Investigation, Validation, Writing – review & editing. ÁB: Writing – review & editing, Data curation, Investigation, Validation. ER-R: Data curation, Investigation, Validation, Writing – review & editing. AV-C: Data curation, Investigation, Validation, Writing – review & editing. GG-R: Data curation, Investigation, Validation, Writing – review & editing. SR-H: Data curation, Investigation, Validation, Writing – review & editing. AL: Data curation, Investigation, Validation, Writing – review & editing. JM: Conceptualization, Formal analysis, Methodology, Supervision, Writing – original draft.
